# rhErythropoietin-b as a tissue protective agent in kidney transplantation: a pilot randomized controlled trial

**DOI:** 10.1186/s13104-014-0964-0

**Published:** 2015-02-03

**Authors:** Beatrice Coupes, Declan G de Freitas, Stephen A Roberts, Ian Read, Hany Riad, Paul EC Brenchley, Michael L Picton

**Affiliations:** Department of Renal Medicine, Manchester Royal Infirmary, Oxford Rd, Manchester, M13 9WL UK; Centre for Biostatistics, University of Manchester, Manchester, M13 9PL UK; Department of Surgery, Manchester Royal Infirmary, Oxford Rd, Manchester, M13 9WL UK

**Keywords:** Ischaemia reperfusion, Delayed graft function, Erythropoietin

## Abstract

**Background:**

Extended criteria donor (ECD) and donation after circulatory death (DCD) kidneys are at increased risk of delayed graft function (DGF). Experimental evidence suggests that erythropoietin (EPO) attenuates renal damage in acute kidney injury. This study piloted the administration of high dose recombinant human EPO-beta at implantation of ECD and DCD kidneys, and evaluated biomarkers of kidney injury post-transplant.

**Methods:**

Forty patients were randomly assigned to receive either rhEPO-b (100,000 iu) (n = 19 in the intervention group, as 1 patient was un-transplantable post randomisation), or placebo (n = 20) in this, double blind, placebo-controlled trial at Manchester Royal Infirmary from August 2007 to June 2009. Participants received either an ECD (n = 17) or DCD (n = 22) kidney. Adverse events, renal function, haematopoietic markers, and rejections were recorded out to 90 days post-transplant. Biomarkers of kidney injury (neutrophil gelatinase-associated lipocalin, Kidney Injury Molecule-1 and IL-18) were measured in blood and urine during the first post-operative week.

**Results:**

The incidence of DGF (53% vs 55%) (RR = 1.0; CI = 0.5-1.6; p = 0.93) and slow graft function (SGF) (32% vs 25%) (RR = 1.1; CI = 0.5-1.9; p = 0.73) respectively, serum creatinine, eGFR, haemoglobin and haematocrit, blood pressure, and acute rejection were similar in the 2 study arms. High dose rhEPO-b had little effect on the temporal profiles of the biomarkers.

**Conclusions:**

High dose rhEPO-b appears to be safe and well tolerated in the early post- transplant period in this study, but has little effect on delayed or slow graft function in recipients of kidneys from DCD and ECD donors. Comparing the profiles of biomarkers of kidney injury (NGAL, IL-18 and KIM-1) showed little difference between the rhEPO-b treated and placebo groups. A meta-analysis of five trials yielded an overall estimate of the RR for DGF of 0.89 (CI = 0.73; 1.07), a modest effect favouring EPO but not a significant difference. A definitive trial based on this estimate would require 1000-2500 patients per arm for populations with base DGF rates of 50-30% and 90% power. Such a trial is clearly unfeasible.

**Trial registration:**

EudraCT Number 2006-005373-22 ISRCTN ISRCTN85447324 registered 19/08/09.

## Background

In 2011-2012, 34% of UK deceased donors were over the age of 60 yrs while 40% were donation after circulatory death (DCD) donors [1]. Both expanded criteria donors (ECD) and DCD kidneys are more likely to develop delayed graft function (DGF) in the early post-transplant period [2,3], with its ensuing clinical and financial implications. Since its introduction, recombinant human erythropoietin has been a major advance in the management of renal anaemia, enhancing patient cognitive function, physical activity and quality of life [4,5]. In addition, there is now a large body of evidence that rhEPO has pleiotropic effects on the body beyond the erythroid compartment. Animal studies examining acute kidney injury, including ischaemia reperfusion injury (IRI) have shown functional improvements [6,7], and anti-inflammatory effects [8] after EPO administration, either before [9,10], during [11,12], or very importantly, after the injury has taken place [6,7,12]. In 2002, Ehrenreich et al. [13] reported a human study in acute ischemic stroke, where high-dose rhEPO was well tolerated and associated with an improvement in outcome at 1 month, as assessed by clinical endpoints of stroke and outcome scales.

Based on results from clinical trials including 1725 patients approximately 8% of patients treated with NeoRecormon® are expected to experience adverse reactions. Undesirable effects are observed predominantly in patients with chronic renal failure or underlying malignancies and are most commonly an increase in blood pressure or aggravation of existing hypertension and headache. The therapeutic margin of NeoRecormon® is very wide. Even at very high serum levels no symptoms of poisoning have been observed [14].

Against this background of evidence supporting the theory that administration of rhEPO after injury may be beneficial, we performed a pilot study to assess the safety of administering high dose rhEPO-beta to ECD and DCD kidney recipients intra- and peri-operatively, to obtain preliminary data on efficacy, and to evaluate changes in three extensively reported biomarkers of kidney injury in blood and urine: NGAL [15,16], IL-18 [17], and KIM-1 [18,19], during the first post-operative week.

## Results

### Patients

**Figure 1:**
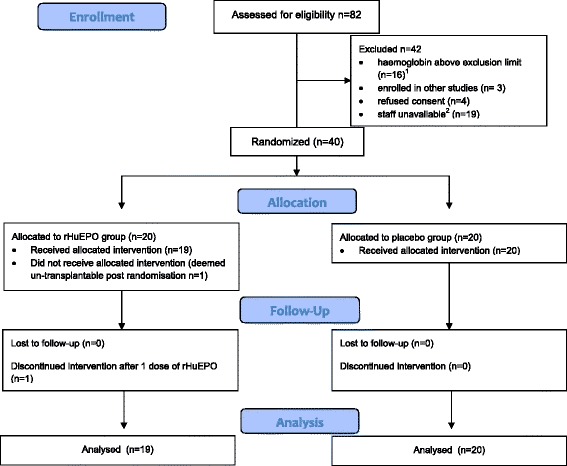
**Screening and randomisation.** ^1^The exclusion limit for haemoglobin was raised to 15 g/dl as a substantial amendment to the protocol with ethical and regulatory authority approval. ^2^The study staff trained in the blinded sequence of delivering the intervention were not always available for out of hours transplants.

Eighty-two recipients were screened (Figure 1) providing 63 eligible candidates 34 donors contributed 39 kidneys, with 5 DCD’s contributing a single kidney into both the rhEPO-b and placebo treated groups. Thirty-nine patients were transplanted, 19 in the rhEPO-b treated group (one patient in the intervention arm was deemed un-transplantable following randomisation), and 20 in the placebo group. No difference was seen in age, sex, ethnicity and BMI of the donor between the groups. Donor cause of death was predominantly an intra-cerebral event in >75% of cases. The rhEPO-b group received more DCD kidneys (63% vs 50%, p = 0.41), particularly DCD kidneys meeting extended criteria (33% vs 20%, p = 0.65). One patient withdrew from the trial following the initial rhEPO-b dose, due to an event attributed to an arterial intimal flap and unrelated to the study drug, but consented to allow continued collection of samples and follow-up data. The patient was included in the final analysis on an intention to treat basis. Demographics for donors and recipients are shown in Table 1. One patient in the rhEPO-b treated group received ATG induction, due to a weakly positive flow cross-match in accordance with transplant unit policy.

**Table 1:** **Demographics of donors and recipients**

***Donors (n = 34)***		
	***rhEPO-b treated n = 19***	***Placebo n = 20***
***Demographics***		
age (yrs) median(IQR)	52 (45-58)	53 (46-66)
male n (%)	10 (53%)	13 (65%)
ethnicity white n (%)	19 (100%)	20 (100%)
***Cause of death n (%)***		
intra-cranial haemorrhage	14 (74%)	14 (70%)
other intra-cranial event	1 (5%)	4 (20%)
extra-cranial event	4 (21%)	2 (10%)
***Donated kidney***		
ECD kidney n (%)	7 (37%)	10 (50%)
DCD kidney n (%)	12 (63%)	10 (50%)
inotropic support n (%)	12 (63%)	16 (80%)
vasopressin n (%)	9 (47%)	11 (55%)
final creatinine (μmol/L) median(IQR)	61(51-87)	77(66-96)
warm ischaemic time^1^ mins (range)	17 (12-22)	17 (13-20)
DCD only		
***Recipients (n = 40)***		
***Demographics***		
age (yrs) median(IQR)	51 (43-63)	54 (41-63)
male n (%)	10 (53%)	14 (70%)
White	13 (68%)	19 (95%)
Asian	5 (27%)	1 (5%)
Afro-Caribbean	1 (5%)	0
***BMI*** *median(IQR)*	25 (23-27)	25 (23-29)
***Cause of ESRD*** *n (%)*		
glomerular disease	8 (42%)	6 (30%)
hypertension	1 (5%)	3 (15%)
reflux nephropathy	4 (21%)	3 (15%)
other	6 (32%)	8 (40%)
***Mode of dialysis pre-transplant*** *n (%)*		
haemodialysis	9 (47%)	12 (60%)
peritoneal dialysis	8 (42%)	7 (35%)
pre-dialysis	2 (11%)	1 (5%)
months on dialysis median(IQR)	30 (16-51)	42 (22-52)
previous transplant n (%)	3 (16%)	5 (25%)
anuric pre-transplant n (%)	6 (32%)	7 (35%)
diabetes n (%)	1 (5%)	0
hypertension n (%)	17 (90%)	17 (85%)
rhEPO pre-transplant n (%)	17 (90%)	17 (85%)
days before transplant of last rhEPO median (IQR)	5 (4-11.5)	4.5 (1.8-11.0)
***PRA%***		
0-5	14	14
6-84	4	5
>84	1	1
***HLA mismatches***		
0	5	4
1-2	5	6
3-4	9	10
***Cold ischaemic time*** hrs.mins (range)	16.52	16.45
	(12.19-32.36)	(11.12-28.42)
***EPO maintenance*** post-transplant n(%)	9 (46%)	11 (55%)
***Blood transfusions*** post-transplant (n)	6	11
***Packed red cell*** post-transplant (units)	1.2 ± 0.5	1.9 ± 0.6
***Acute rejection episodes*** in 3 mths post-transplant	5	3

^1^warm ischaemic time in DCD was defined as the time from asystole to in-situ cold perfusion.

IQR = interquartile range, BMI = body mass index, ESRD = end stage renal disease, PRA = panel reactive antibodies.

### Pre-transplant dialysis

In the rhEPO-b group 5/19 received 2 hours of haemodialysis and one patient received rapid cycling peritoneal dialysis with varying degrees of ultrafiltration immediately prior to surgery. Of these, four patients subsequently developed DGF. In the placebo group 4/20 received 2 hours of haemodialysis prior to surgery and all developed DGF. Of interest, 17/21 patients on maintenance haemodialysis prior to surgery developed DGF, as opposed to 3/15 patients on maintenance peritoneal dialysis (RR 4.05; CI 1.44-11.38; p = 0.0005).

### Adverse events

There were two deaths due to sepsis in the placebo group. Hypertension occurred in 6 rhEPO-b recipients and 5 patients in the placebo group (RR = 0.79; CI = 0.29-2.17; p = 0.73). One patient in the rhEPO-b group developed a generalised tonic-clonic seizure due to severe hypertension, attributed to hypervolaemia and the cessation of anti-hypertensive medication peri-operatively (unit policy). In the opinion of the respective attending consultants and the independent trial data monitor, none of the observed adverse events was attributable to rhEPO-b treatment.

### Graft function early post-transplant

Numbers and duration of dialysis episodes were similar in both groups (Table 2). DCD kidney recipients were more likely to develop DGF (15/22) than ECD kidney recipients (5/17) (RR = 2.47; CI = 1.13-5.39; p = 0.01). Seven of 12 DCD recipients in the rhEPO-b group and 8/10 in the placebo group developed DGF (RR = 1.37; CI = 0.77-2.42; p = 0.38). Similarly 2/7 ECD recipients in the rhEPO-b group and 3/10 in the placebo group developed DGF (RR = 1.05; CI = 0.23-4.73; p = 1.0). The most common indication for first dialysis was hyperkalaemia.

**Table 2:** **Graft function and dialysis early post-transplantation**

	***rhEPO-b group (n = 19)***	***Placebo group (n =20)***	**RR (CI) p**
*Primary non-function*	0	0	
*Delayed graft function n (%)*	10 (53%)	11 (55%)	RR = 1.0 (0.5-1.6) p = 0.93
*Slow graft function n (%)*	6 (32%)	5 (25%)	RR = 1.1 (0.5-1.9) p = 0.73
Days to first dialysis*	1 (1-3)	1 (1-3)	
Number of dialysis episodes*	3 (1-17)	4 (1-19)	
Days to last dialysis*	7 (1-41)	8 (1-35)	

*median (IQR).

### Graft function out to 90 days post-transplant

**Figure 2:**
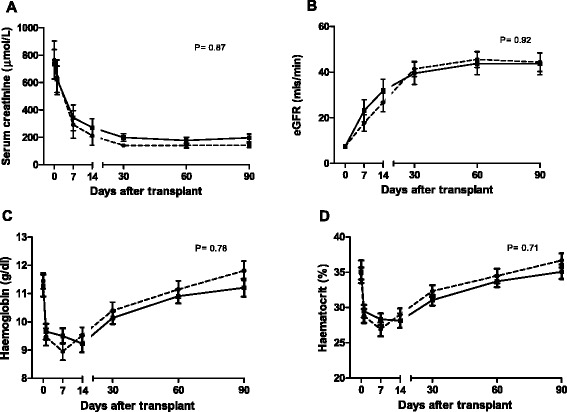
**Sequential serum creatinine, 4-variable MDRD eGFR, haemoglobin and haematocrit profiles. (A)** serum creatinine and **(B)** eGFR (4v MDRD). Renal function was similar in the EPO and placebo treated patients, with no significant differences. **(C)** Haemoglobin and **(D)** Haematocrit during the first 3 months post-transplant. Haematocrit levels were similar in the EPO and placebo treated patients with no significant differences. EPO treated group placebo group Data expressed as mean ± SEM, ANOVA.

Sequential serum creatinine and 4-variable MDRD eGFR are shown in Figure 2A and B respectively. Kidney function (eGFR) was not analysed prior to day 7, apart from baseline pre-transplant, due to the high rate of DGF and the impact of dialysis, preventing meaningful analysis. Acute rejection rates were similar in the rhEPO-b treated and placebo groups during the first 3 months post-transplant (5 vs 3; RR = 0.57; CI = 0.16-2.1; p = 0.45).

### Haematological parameters

Haemoglobin levels were similar in the rhEPO-b and placebo groups on entry into the study (11.5 ± 0.3 g/dl *vs* 11.3 ± 0.4 g/dl, respectively; p = 0.78). The number of blood transfusions required during the in-patient stays did not differ significantly between groups (p = 0.20) and the groups had similar levels of maintenance rhEPO-b usage post- transplant (Table 1). There was no effect on platelet levels at any time point (data not shown). Haemoglobin and haematocrit profiles are shown in Figure 2C and D respectively, showing no significant differences between the groups.

### Biomarkers

**Figure 3:**
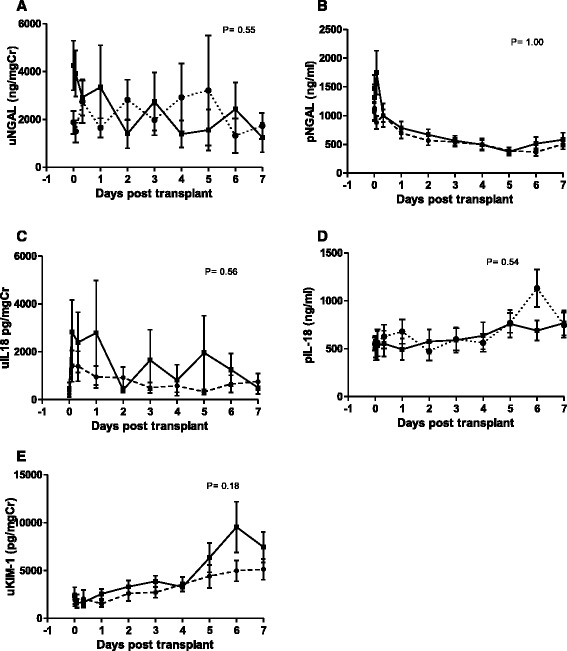
**Biomarkers of kidney injury (A) uNGAL ng/mgCr (B) pNGAL (ng/ml) (C) uIL-18 pg/mgCr (D) pIL-18 (ng/ml) (E) uKIM-1 pg/mgCr.** rhEPO treated group placebo group Data shown as means +/- SEM. P values are for an overall difference between EPO and placebo treated, based on a mixed-effect ANOVA model.

The temporal profiles in blood and urine of the biomarkers of renal injury, neutrophil gelatinase-associated lipocalin (NGAL), Kidney Injury Molecule-1 (KIM-1) and, IL-18 are shown in Figure 3A-E. There were small differences between the rhEPO-b and placebo treated groups, none of which reached statistical significance.

### Meta-analysis of 5 trials of rhEPO in transplantation

**Figure 4:**
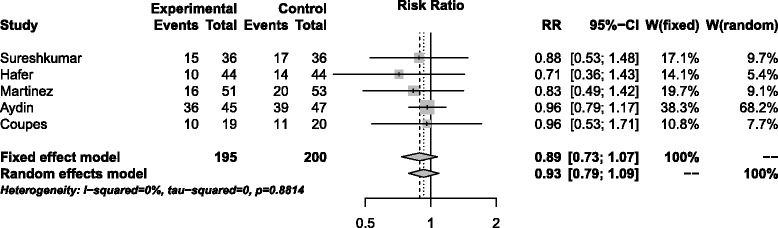
**A meta-analysis of 5 randomised controlled trials of the effect of high dose EPO on DGF represented as a Forest plot.**

A meta-analysis (Figure 4) including data from this trial and from those described by Sureshkumar et al. [20], Hafer et al. [21], Martinez et al. [22] and Aydin et al. [23] yielded an overall estimate of the RR for DGF of 0.89 (CI = 0.73; 1.07), a modest effect favouring rhEPO, but not demonstrating a significant difference between rhEPO and placebo treatments.

## Discussion

This pilot study supported the view that the intra- and peri-operative intra-venous administration of high dose rhEPO-b (a total infusion of 100,000 iu of rhEPO-beta) appeared to be safe in the early post-transplant period. The study reports only small effect sizes of rhEPO-b on adverse events, renal function, haematopoietic factors, acute rejection episodes, and the profiles of biomarkers of kidney injury post-transplant.

The dose regimen in our study was adapted from Ehrenreich et al. [13], where high levels of EPO were required to cross the blood-brain barrier to provide a high concentration in the cerebrospinal fluid. In renal patients, the maximum recommended dose in renal failure is 720 iu/kg/week [24], which in the average 70 kg patient, is half the dose administered in this study. Furthermore, mathematical modelling in healthy volunteers has demonstrated that a similar dose (1000 U/kg) resulted in >98% occupation of EPO receptors, which persisted for 2 days following the dose [25]. The majority of the recipients in this study were receiving maintenance rhEPO for anaemia associated with end-stage renal failure, though administration of rhEPO stopped more than 2 days prior to transplant in all but 7 recipients. It is conceivable that tissue protection was afforded by low levels of rhEPO to both groups, and thus confounded the outcome, with a dose ceiling effect for EPO. However, the retrospective review from Mohiuddin et al. [26] of patients receiving anaemia maintenance doses of rhEPO at the time of transplantation found no difference in the DGF rate or haemoglobin levels compared to those patients not receiving rhEPO, out to three months post-transplant.

Song et al. [27] administered 300 mg/kg of erythropoietin-beta IV to 36 adults undergoing coronary artery bypass grafting at induction of anaesthesia and noted a reduction in the incidence of acute kidney injury (EPO 8% vs Placebo 29%, p = 0.03), defined as a 50% increase in serum creatinine over baseline in the first 5 post-operative days. However Poulsen et al. [28] examined the effect of high dose EPO (500 iu/kg IV 12-18 hrs pre-op and at induction) in patients undergoing coronary artery bypass with no apparent difference between treatment and placebo groups with regard to serum creatinine. Martinez reported a French multicentre placebo controlled trial examined the effect of 40,000U EPO IV administered before kidney transplantation, at 12 hours, 7 days and 14 days post-operatively on the incidence of DGF [22]. There was no difference in DGF rates between the groups (EPO 32% vs placebo 29%), with similar eGFRs at 1 month post-implantation. A retrospective study in France compared recipients on 250 U/kg/week of EPO at the time of transplantation to recipients not receiving EPO and found no difference in graft function or haemoglobin level at 1 month [29]. Furthermore, the German multi-centre EPO Stroke Trial, a Phase II/III trial designed to reproduce the promising results of the earlier EPO Stroke Study (improved clinical recovery in EPO treated patients with ischemic stroke), was a negative trial that also raised safety concerns [30]. Aydin et al. [23] reported a 12-month, randomized, double-blind, placebo- controlled trial of high-dose recombinant human erythropoietin-b (Epoetin) in 92 donation after cardiac death kidney transplant recipients. Patients were randomized to receive an intravenous bolus of Epoetin (3.3 × 10^4^ IU; n = 45) or placebo (saline 0.9% solution; n = 47) on 3 consecutive days, starting 3–4 h before the transplantation and 24 h and 48 h after reperfusion. Results showed no differences in the incidence or duration of delayed graft function and/or primary non-function (Epoetin 77.8 vs. placebo 78.7%, p = 1.00) though Epoetin treatment significantly increased the risk of thrombotic events at 1 month and 1 year (Epoetin 24.4% vs. placebo 6.4%, p = 0.02).

In patients with an acute ST-segment elevation myocardial infarction a single intravenous bolus of EPO-alpha within 4 hours of percutaneous coronary intervention did not reduce infarct size [31].

Two other randomised controlled trials investigating high dose EPO in renal transplantation have been published recently. Sureshkumar et al. [20] conducted a randomised double blind clinical trial where the primary end point was the level of graft function in the early post-transplant period. Thirty-six patients in each group were included in the final analysis and their data gave similar event rates to the study reported here, with a RR for DGF of 0.88 (0.53-1.48). There were no clinically demonstrable beneficial effects of high dose EPO-alpha given intra-arterially during the early reperfusion phase in deceased donor kidney recipients, in terms of reducing the incidence of DGF or improving short-term allograft function. The authors also measured two biomarkers (NGAL and IL-18) post transplantation and found similar levels in the EPO treated group versus the control group. They did not report an increase in adverse events in the EPO treated group.

In the second study Hafer et al. [21] evaluated high dose EPO-alpha administered intra-and post-operatively to recipients of deceased donor kidneys, with a primary study end point of allograft function 6 weeks post-transplant. The study recruited 45 patients to each arm. There was no significant effect of EPO-alpha on either long-term graft function (eGFR at 12 months) or histology (protocol biopsies at 6 weeks and 6 months), with a RR for DGF again similar to our study at 0.71 (0.36-1.43), but with a lower overall event rate of 27%.

The doses of rhEPO used by Sureshkumar et al. (40,000 iu rhEPO-alpha injected into the iliac artery) and Hafer et al. (3 doses of 40,000 iu rhEPO-alpha) were similar to our study (3 doses of 33,000 iu rhEPO-beta). It is conceivable that a lack of efficacy is a consequence of the timing of EPO administration. Injury prior to reperfusion is multi-factorial and due to recurrent insults to the kidney rather than a single ischaemia reperfusion event. Typically injury begins with peri-mortem events including, severe hypertension or hypotension and nephrotoxic agents. Retrieval of the kidneys is associated with warm ischaemia, cold ischaemia and the anastomotic ischaemic phase. The lack of efficacy in these studies may be due to the delay in rhEPO-b administration until reperfusion. However, therapeutic intervention in the donor, prior to organ retrieval and storage, would impact on all organs donated and prevent any potential recipients from declining involvement in the study without declining organ transplantation. It was not possible to intervene during cold storage firstly because we did not have access to machine perfusion technology at the time, and secondly the potential recipient would not have been able to decline involvement in the study. We decided to intervene immediately prior to reperfusion, with the knowledge that significant injury to the kidney may have already occurred, but based on the experimental evidence, intervention could still be beneficial to the graft. The participants in our study received kidneys from DCD and ECD donors. In the study from Sureshkumar et al. the kidneys were from ECD donors in only 46% of the cases. The donor details were not presented in the study from Hafer et al., though their exclusion criteria included immunological loss of a previous graft, and cold ischaemic time of longer than 24 hours.

## Conclusions

Taking the five studies together, a range of quality of donated kidneys has been examined and the evidence appears convincing that high dose rhEPO in deceased kidney transplants, although well tolerated, has little effect on DGF, early or later allograft function, graft histology, or levels of biomarkers of kidney injury, including NGAL, IL-18 and KIM-1. Additionally, concerns were raised over the increased number of thrombotic events in the EPO treated groups in some of the trials. A meta-analysis of the five trials yielded an overall estimate of the RR for DGF of 0.89 (CI = 0.73; 1.07), a modest effect favouring EPO but not demonstrating a significant difference. At a time when there is obvious need to maximise the lifespan of donated organs it is disappointing that the promising experimental evidence of rhEPO tissue protection does not readily translate into the clinical setting of transplantation.

## Methods

### Ethical approval

The study was conducted in accordance with the ethical principles of the Declaration of Helsinki and was consistent with the International Conference on Harmonization of Good Clinical Practice. The clinical and research activities reported are consistent with the Principles of the Declaration of Istanbul as outlined in the Declaration of Istanbul on Organ Trafficking and Transplant Tourism. The study was approved by the Central Manchester Research Ethics Committee (07/Q1407/94), the Medicines for Human use Regulatory Authority (EuDract no. 2006-005373-22), and registered with the ISRCTN (number 85447324; 19/08/09). Informed consent was obtained from each patient. The manuscript adheres to CONSORT guidelines for reporting clinical trials [32].

### Patients

Patients were eligible if aged 18 years or more, able to give consent, and in receipt of a Maastricht category III, (awaiting cardio-circulatory death after withdrawal of treatment), a Maastricht category IV (cardio-circulatory death in a brain dead donor), a kidney from an extended criteria donor (defined as equal to or greater than 60 years old, or 50-59 years with combinations of cerebrovascular accident, hypertension or serum creatinine greater than 133 μmol/l, or a kidney with a cold ischaemic time greater than 24 hours). Exclusion criteria included inability to consent, pregnancy, breastfeeding, acute infection, previous intolerance of the trial drug, a diastolic blood pressure > 100 mm/Hg pre-transplantation, or initially a haemoglobin level = or > 13 g/dl. However, it became clear that a haemoglobin cut-off of 13 g/dl would exclude approximately one third of otherwise eligible participants. A review of the pre-operative haemoglobin levels of adults transplanted at this centre in 2007 reported a mean pre-op haemoglobin of 12.2 g/dl, with a range of 7.4-17.7 g/dl, and a mean decrease of 2.4 g/dl within 24 hours of surgery. As a result, the haemoglobin exclusion criterion was reset to ≥15 g/dl, as a substantial amendment to the protocol with ethical and regulatory authority approval. All patients received immunosuppression as per unit protocol. Induction immunosuppression consisted of basiliximab 20 mg intravenously on day 0 and day 4, as well as a single dose of methylprednisolone 1 g given intra-operatively. Maintenance immunosuppression consisted of tacrolimus (Prograf®), prednisolone and mycophenolate mofetil (Cellcept®). DGF was defined as the need for haemodialysis or peritoneal dialysis within the first seven days post transplantation, and slow graft function was defined as a creatinine reduction ratio at day 7 of <70% [33].

### Randomisation and blinding

Eligible patients were randomly assigned by the trial Pharmacy using a computer-generated list to receive either rhEPO-b (Neorecormon® Roche) or 0.9% saline. All study participants and the clinical team were blinded to the trial drug for the duration of the study. Pharmacovigilance was undertaken by PDMS Roche Products Limited.

### Sample collection and processing

The first dose of rhEPO-b (33,000 iu) (Neorecormon® Roche) or 0.9% saline were administered intra-operatively in a 100 ml intra-venous infusion over 15 minutes, as clamps were released to allow graft reperfusion. The second and third infusions (each 33,000 iu) were given at 24 hour intervals post-operatively, making a total rhEPO-b dose of approximately 100,000 iu over approximately 48 hours. Prior to reperfusion, a 20 ml systemic blood sample was collected via the central line (the pre-reperfusion sample). The post-reperfusion 20 ml blood sample was collected approximately 15 minutes following reperfusion. 10 ml blood samples were collected at 2, 8 and 24 hours, and daily for the first 7 days post-operatively, coinciding with venepuncture for routine care where possible. Blood samples were centrifuged at 2000 rpm for 10 minutes to separate the plasma, which was aliquoted and stored at -80C. Urine samples were collected at similar time points where possible, centrifuged at 2000 rpm for 5 minutes, aliquoted and stored at -80C. Samples were batched for analysis of biomarkers.

### Measurement of biomarkers

Plasma NGAL (pNGAL) and urine NGAL (uNGAL) were measured by immunoassay using the Duoset DY1757, and urine KIM-1 (uKIM-1) using the duoset DY1750, R&D Systems, OXON UK, following the manufacturer’s guidelines. Mature IL-18 in plasma and urine was measured using an in-house immunoassay (capture antibody - Clone 125-2H, detection antibody - Clone 159-12B, and standard - rHuIL-18, MBL, Medical and Biological Labs co. Ltd, Nagoya, Japan). Sample concentrations in all assays were calculated from a 4-parameter standard curve (SOFTmax PRO v4 software, Molecular Devices, Ca 94089). Biomarkers in urine were corrected for creatinine concentration in the sample. An internal standard was included on each assay plate confirming an inter-assay coefficient of variation (CV) of <20% and an intra-assay CV of <10%.

### Sample size and statistical analysis

As a pilot, the study had no formal power calculation, but was designed to test the safety of delivering the intervention, to describe effect sizes on the measured variables (adverse events, renal function, haematopoietic markers, and acute rejection episodes), and to evaluate the profiles of three biomarkers of kidney injury. A sample size of 20 per arm was chosen on the basis of feasibility and to gather sufficient data to design and power a definitive trial [34].

Data analysis was performed using GraphPad Prism 5. Results were presented as median ± interquartile range, or as percentages as appropriate. Mann-Whitney U tests were used to compare continuous variables between groups. Categorical data was analysed using a Fisher’s exact test to generate a risk ratio (RR) and confidence interval (CI). Clinical and biomarkers assessed at multiple times were compared using mixed-effect ANOVA models to allow for correlations between repeat measures using the lmer package in the R statistical environment [35]. A meta-analysis including this trial and those described by Sureshkumar et al. [20], Hafer et al. [21], Martinez et al. [22] and Aydin et al. [23] was conducted to derive a pooled RR estimate using the Mantel-Haenszel estimator and a random effects estimator, and the meta package in R. A two- tailed significance level of 0.05 was used throughout.

## Abbreviations

DCD: Donation after circulatory death
DGF: Delayed graft function
ECD: Extended criteria donor
IRI: Ischaemia reperfusion injury
KIM-1: Kidney injury molecule-1
NGAL: Neutrophil gelatinase-associated lipocalin
rhEPO-b: rhErythropoietin-beta
SGF: Slow graft function
